# A Little Switch: Alternative Domain Conformations Control Bacterial Flagella Rotation Direction

**DOI:** 10.1371/journal.pbio.1001480

**Published:** 2013-02-12

**Authors:** Richard Robinson

**Affiliations:** Freelance Science Writer, Sherborn, Massachusetts, United States of America

**Figure:**
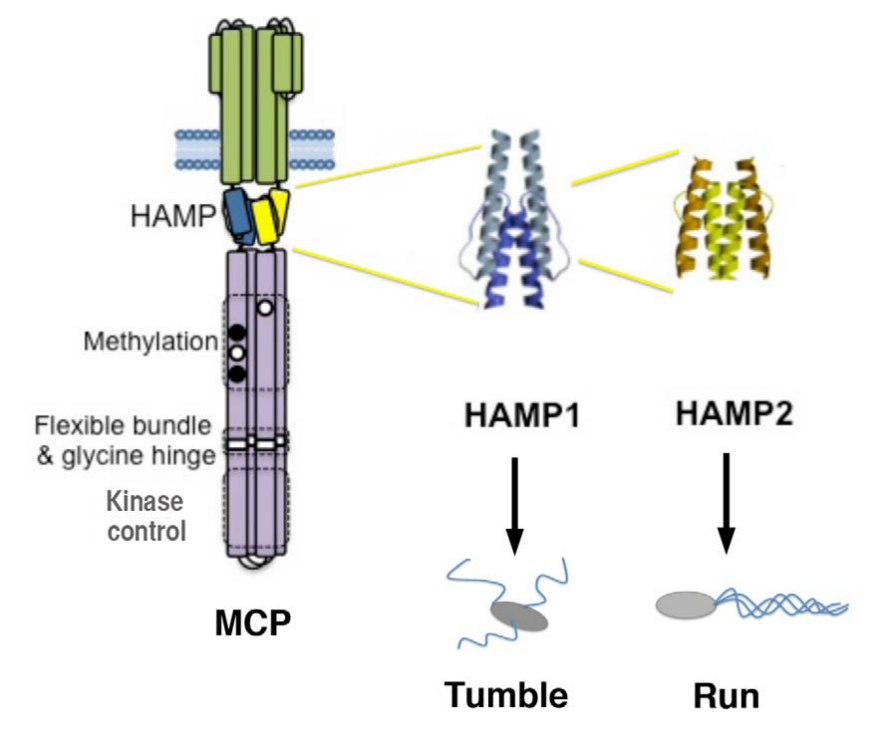
HAMP domains of bacterial chemoreceptors (MCPs) switch from one conformation (HAMP1) to another (HAMP2) to relay the binding of signaling molecules outside the cell (top) to regulatory circuits within the cell (bottom), thereby flipping the direction of flagellar rotation. HAMP1 and the bacterial cell tumbles; HAMP2 and it runs. Image credit: Joe Jones.

The flagella of *E. coli* move the bacterium in one of two ways. When they spin counterclockwise, the cell is propelled forward in a straight line. When they spin clockwise, the bacterium tumbles in place, ultimately pointing in some new, random direction, ready for another straight-line run.

The search for food is a major trigger for this tumbling and running, and the direction of flagellar spin is regulated in part by methyl-accepting chemotaxis proteins (MCPs). The extracellular domain of an MCP is a chemosensor, which responds to changing concentrations of its target molecule by a shape change.

The signal embodied by that shape change is transduced first through a membrane-spanning domain, then to one or more small linkers called HAMPs, and finally to a kinase control module, which can turn on, or turn off, a kinase, which then either does, or doesn't, phosphorylate another protein. The ratio of the phosphorylated to unphosphorylated protein is the final determinant of the direction of flagellar rotation, and hence the movement of the bacterium.

The HAMPs are not merely passive links in this chain; instead, they are believed to relay the signal from the chemosensor to the control module. How they do so has now been elucidated by Michael Airola, Brian Crane, and colleagues, who show that by adopting alternative conformations, the HAMP domain acts like a switch to turn on or turn off kinase activity, and cause the bacterium to tumble or run.

The authors knew that some bacterial proteins contained HAMPs of two different shapes. Both were made of four helices. While in one (HAMP1) the helices were lined up alongside each other, in the other (HAMP2), they were distorted. One possible explanation was that the two structures were snapshots of a single structure that could flip back and forth between the two conformations.

To explore this question, they constructed a chimeric receptor and expressed it in *E. coli*, using a HAMP from another bacterium that is more easily characterized structurally. They used a technique called pulsed-dipolar electron spin resonance spectroscopy to make measurements on the proteins in solution, and employed various genetic mutants to lock the structures in place. They found that HAMP1 and HAMP2 were indeed alternative conformations, and that they send opposing signals to the kinase control module, leading to opposing flagellar movements. They identified a key residue in the HAMP structure that stabilized the inhibitory state, and showed that mutation of this residue could flip the HAMP into the alternative conformation and “turn on” the activity of the protein.

HAMPs are found in over 26,000 receptor proteins in bacteria, Archaea, and simpler eukaryotes. HAMP itself is an acronym for the four major output domains that the HAMPs connect to: histidine kinases, adenylyl cyclases, methyl-accepting chemotaxis proteins, and phosphatases. The number and variety of HAMP-containing proteins suggest that the structural switching mechanism identified here is also likely to be employed in many other proteins and in other species. But it is not likely to be universal, the authors argue, since the very plasticity of the HAMP domain means it may also be amenable to other kinds of structural changes serving other kinds of functions.

Finally, the authors note that understanding how to manipulate HAMP structure to alter the output of chemotaxis proteins may have some practical uses. The wide variety of metabolic pathways employed by bacteria make them invaluable for cleaning up a host of toxic substances, such as those in chemical spills, but in some cases their normal chemotactic response may be to avoid high concentrations of the toxin. It may be possible to bioengineer bacteria to seek out such toxins, tweaking their chemosensory apparatus so they run toward, rather than away from, the spill.

**Airola MV, Sukomon N, Samanta D, Borbat PP, Freed JH, et al. (2013) HAMP Domain Conformers That Propagate Opposite Signals in Bacterial Chemoreceptors. doi:10.1371/journal.pbio.1001479**

